# Reimagining spaces where children play: developing guidance for thermally comfortable playgrounds in Canada

**DOI:** 10.17269/s41997-021-00522-7

**Published:** 2021-06-15

**Authors:** Eric Kennedy, Heather Olsen, Jennifer Vanos, Daniel J. Vecellio, Marla Desat, Karina Richters, Alexandra Rutledge, Gregory R. A. Richardson

**Affiliations:** 1 National Program for Playground Safety, Cedar Falls, IA USA; 2 Biomedical Engineering Department, Bucknell University, Lewisburg, PA USA; 3 Department of Health, Recreation, and Community Services, University of Northern Iowa, Cedar Falls, IA USA; 4 School of Sustainability, Arizona State University, Tempe, AZ USA; 5 Department of Geography, Texas A&M University, College Station, TX USA; 6 Strategy and Stakeholder Engagement Branch, Standards Council of Canada, Ottawa, ON Canada; 7 Environmental Sustainability and Climate Change, City of Windsor, Windsor, ON Canada; 8 Climate Change and Innovation Bureau, Health Canada, Ottawa, ON Canada

**Keywords:** Child, Playground, Microclimate, Environment, Environment and public health, Recreation, Enfants, terrain de jeux, microclimat, environnement, environnement et santé publique, récréation

## Abstract

**Setting:**

Planning and designing thermally comfortable outdoor spaces is increasingly important in the context of climate change, particularly as children are more vulnerable than adults to environmental extremes. However, existing playground standards focus on equipment and surfacing to reduce acute injuries, with no mention of potential negative health consequences related to heat illness, sun exposure, and other thermal extremes. The goal of this project was to develop proposed guidelines for designing thermally comfortable playgrounds in Canada for inclusion within the CAN/CSA-Z614 Children’s playground equipment and surfacing standard.

**Intervention:**

The project to develop guidance for thermally comfortable playgrounds was initiated with a municipal project in Windsor, Ontario, to increase shade, vegetation, and water features at parks and playgrounds to provide more comfortable experiences amid the increased frequency of hot days (≥30°C). The lack of available information to best manage environmental conditions led to a collaborative effort to build resources and raise awareness of best practices in the design of thermally comfortable playgrounds.

**Outcomes:**

A group of multidisciplinary experts developed technical guidance for improving thermal comfort at playgrounds, including a six-page thermal comfort annex adopted within a national playground and equipment standard. The annex has been used by Canadian schools in a competition to design and implement green playgrounds.

**Implications:**

Both the technical report and the thermal comfort annex provide increased awareness and needed guidance for managing environmental conditions at playgrounds. Thermally safe and comfortable play spaces will help ensure that Canada’s playgrounds are designed to minimize environmental health risks for children.

## Résumé

**Contexte:**

À l’ère des changements climatiques, le confort thermique n’a jamais été si important pour la planification et l’aménagement d’espaces extérieurs, surtout car les enfants sont plus sensibles que les adultes aux chaleurs brûlantes. Cependant, les normes qui encadrent actuellement les aires de jeu s’articulent autour d’une logique de prévention de traumatismes. Elles portent donc principalement sur les équipements et leurs revêtements plutôt que sur les conséquences des canicules, des malaises liés à la chaleur et des insolations. L’objectif du présent projet est de rédiger un projet de directives pour favoriser le confort thermique des aires de jeu au Canada et d’intégrer ces directives à la norme CAN/CSA-Z614 Aires et équipements de jeu.

**Intervention:**

Les origines du projet remontent à une action municipale à Windsor, en Ontario. Celle-ci avait pour but d’augmenter la quantité de zones d’ombres, de végétation et de jeux d’eau dans les parcs et les aires de jeu en vue de favoriser le confort thermique vu la fréquence croissante des canicules (≥30°C). Le constat de lacunes d’informations autour de la gestion des conditions écologiques a suscité une concertation pour créer des ressources et faire rayonner des pratiques d’aménagement optimales pour l’amélioration du confort thermique des aires de jeu.

**Résultats:**

Une équipe transversale d’experts a rédigé des directives techniques pour améliorer le confort thermique des aires de jeu, ainsi qu’une annexe de six pages sur la question qui a été intégrée à une norme nationale. Dans le cadre d’un concours, des écoles canadiennes se sont servies de l’annexe comme référentiel pour penser et aménager des aires de jeu vertes.

**Implications:**

Le rapport technique et l’annexe mettent en valeur le besoin d’encadrer la gestion des facteurs environnementaux des aires de jeu. L’amélioration du confort thermique de ces espaces et la réduction des risques environnementaux permettent de protéger la santé des enfants au Canada.

## Introduction

Playgrounds are found in communities across Canada and provide children with healthy spaces to play. Playing in outdoor spaces, especially in nature, can increase children’s physical activity levels (Kneeshaw-Price et al., 2013; Schaefer et al., 2014; Lachowycz et al., 2012), decrease childhood obesity (Herrington & Brussoni, 2015), increase attention (Grahn et al., 1997), and reduce stress levels (Wells & Evans, 2003). However, while outdoor play is an essential ingredient for positive child health, playgrounds are the leading cause of recreational injury for children 1 to 10 years of age (Schwebel & Brezausek, 2014; Canadian Institute for Health Information, 2016).

Historically, playground injury prevention initiatives have focused on improving playground equipment and surface materials, including installation and maintenance practices (Ball, 2004). Other health risks at playgrounds, like heat stress, have not been tracked or researched to the same extent. It is known that children are more vulnerable than adults to hot ambient environments and heat illness symptoms (Sinclair et al., 2007) and are more susceptible to sunburns and thermal burn injuries because of their more sensitive skin (Volkmer & Greinert, 2011). Furthermore, Canadian skin cancer rates are rising, which are often linked to childhood or adolescent sun exposure or sunburns (Canadian Cancer Society, 2014).

Environmental conditions at playgrounds related to sun and heat exposure warrant increased attention for potential harmful health consequences. For example, many playgrounds lack adequate shade provisions (Olsen et al., 2019), and contemporary playgrounds with artificial materials and little shade have some of the highest surface temperatures within an urban area (Vanos et al., 2016). Uncomfortable thermal conditions at playgrounds can affect the number of visitors, duration of visits, and activity levels of play (Semenzato et al., 2011; Vanos et al., 2017a). Researchers have called for a change in policies and regulations to improve warm-season thermal comfort through design to ensure that children can safely and effectively play outdoors (Vanos et al., 2016; Hyndman, 2017).

Planning and designing thermally comfortable outdoor spaces is increasingly important in the context of climate change. The number of extremely hot days in Canada (≥30°C) is expected to become more common because of climate change, with negative impacts to human health (Berry et al., 2014). For example, in Windsor, Canada’s southernmost city, the number of extreme days where the temperature exceeds 30°C is expected to double by 2050 from the 1976–2005 reference period (Climate Atlas of Canada, 2018). There is a need to improve the design of playgrounds in Canada to ensure children can safely play under these warmer conditions.

A standard is a consensus-built document designed to provide guidance, definitions, or minimum technical requirements. The CSA Group publishes and maintains the standard CAN/CSA Z614 *Children’s playground equipment and surfacing* which provides requirements for public-use playground equipment to minimize the likelihood of life-threatening or serious injuries (CSA Group, 2020). Even though CAN/CSA Z614 is a voluntary standard, it can be made mandatory through references in regulations, in procurement and eligibility requirements, and through policies. For example, both the Governments of Ontario and Quebec require provincially regulated child care centres to ensure that any outdoor play space and associated play equipment comply with the requirements of the standard (Ontario, 2014; Quebec, 2020).

Previous editions of the CAN/CSA-Z614 standard have not included design and management considerations for extreme weather conditions, particularly heat, humidity, cold, or wind. Furthermore, there are minimal informational resources available which address environmental hazards to improve thermal comfort (Madden et al., 2018; Brown, 2010), and no materials address the Canadian climate in relationship to playgrounds. To help address the lack of information on this topic, the CSA Group, Health Canada, the Standards Council of Canada (SCC), and the National Program for Playground Safety (NPPS) collaborated on a project to integrate advice for designing thermally comfortable playgrounds within CAN/CSA Z614. The goal of the project was to ensure playgrounds are designed to be thermally safe year round, especially during periods of extreme heat in summer, to maximize the health and social benefits of longer and more active play.

## Intervention

This project aimed to generate guidance on designing thermally comfortable playgrounds for all seasons of play in Canada and propose changes to the next edition of the CAN/CSA-Z614 standard. It evolved over a 7-year period in the following key stages.

### Pilot project: improving thermal comfort in City of Windsor parks and playgrounds

In 2013, the City of Windsor, with support from Health Canada, conducted a study to investigate thermal comfort in six of Windsor’s parks and playgrounds (Blanchard, 2013). Using satellite imagery and infrared cameras, the City discovered that rubber mats under playground equipment reached over 70°C, temperatures hot enough to cause a 1st or 2nd degree burn (ISO 13732, 2010). The report recommended various actions, including developing shade guidelines and changes to the design of parks and playgrounds to include cool features such as water fountains, trees, lighter-coloured rubber mats, splash pads, and shade structures.

At the conclusion of the 2013 study, the City of Windsor began to physically implement improvements to parks and playgrounds by increasing the availability of shade, planting vegetation, and installing water features (e.g., splash pads, drinking fountains) to help increase visitor comfort, satisfaction, and utilization of these spaces (Health Canada, 2020). Overall, 13 municipal parks and 27 playgrounds have been modified since this project began. It was during this project that the lack of available information for designing thermally comfortable playgrounds was identified. Windsor’s experience has provided valuable lessons learned that helped inspire action to update the CAN/CSA Z614 standard and provide clear guidance to playground designers across Canada.

### Pitching the idea to the Canadian Standards Association

As a federal crown corporation responsible for the standardization system in Canada, SCC accredits CSA Group to develop standards according to international best practices. In 2016, the SCC launched the *Standards to Support Resilience in Infrastructure Program*, a 5-year program to integrate climate adaptation considerations in new and updated standards and guidance for buildings and infrastructure.

The SCC in 2016 became aware of the foundational work undertaken in Windsor, with support from Health Canada, and the potential for further work to integrate thermal comfort and extreme heat considerations into the CAN/CSA-Z614 standard. In 2018, SCC facilitated a discussion between Health Canada and CSA Group regarding a short informational annex for city planners, designers, and practitioners for integrating thermal comfort into playground designs, in the context of climate change. Following the meeting, CSA Group confirmed its interest in receiving an advisory report prepared by experts with recommendations on how best to integrate thermal comfort considerations within the CAN/CSA-Z614 standard. In early 2019, the SCC commissioned the NPPS to review the literature, gather information from topical experts, and develop a report that consolidates best practices in thermally comfortable playground design.

### Thermal comfort needs assessment

In support of developing a technical report, the NPPS in 2019 gathered perspectives about thermal comfort and children’s playgrounds through a comprehensive needs assessment. The work involved a survey of a diverse group of 80 experts based in communities across Canada. Invited survey participants included professionals in public health, education, environmental science, landscape architecture, engineering, and urban planning. The survey was deployed in both English and French languages. The overall response rate was 69% (55) of the invited participants, with 91% of respondents (50) having professional expertise in the Canadian context, covering all geoclimates of Canada.

The focus of the survey was to collect information on thermal comfort and playgrounds. The survey gathered participant perspectives on: environmental factors which influence design priorities and safety elements, perceptions and contributions of thermal comfort, and mitigation strategies associated with thermal comfort management. The survey found that thermal comfort was universally recognized as an important element in children’s playgrounds (97%), yet there was a strong consensus that these environmental factors have not been integrated into playground designs, as compared with playground safety factors addressed within the previous version of the standard (e.g., materials, structural integrity, surfacing, inspection, maintenance). Additionally, the survey found that topics addressed within the standard as informative annexes (e.g., accessibility, design for supervision, and supervision practices) were much more likely to receive design priority than shade, water features, or other issues related to thermal comfort.

## Outcomes

This project has resulted in three important outcomes that help promote the design of playgrounds where children can enjoy safe and active play in all environmental conditions.

### Thermally comfortable playgrounds: a review of literature and survey of experts

The NPPS developed a detailed report to provide contextual knowledge of the impacts of environmental conditions on children’s usage of play spaces and recommendations for integrating thermal comfort into the CAN/CSA-Z614 standard (Kennedy et al., 2020). The report raises awareness about hazardous thermal conditions at playgrounds and provides policy and design considerations for improving thermal comfort for children at playgrounds. It also provides actionable design considerations to improve thermal comfort for all seasons of play. Guidance in the report drew heavily from both the academic and grey literature about playgrounds and thermal comfort, as well as survey results from the diverse group of experts consulted. The report identified four principal environmental factors within playgrounds: solar radiation, temperature, relative humidity, and airflow—it also addressed the benefits of accessible water features (Table 1).

**Table 1:** Environmental factors that influence the thermal comfort of playgrounds along with a description of influencing features

Environmental factors	Effect on thermal comfort	Influencing design elements
Solar radiation	- Critical determinant of thermal discomfort in warm season - May cause extreme surface temperatures, leading to thermal burns	Natural shade - Deciduous trees provide shade in summer and let sunlight through in winter - Coniferous trees provide year-round shade; can block cold winter winds Manufactured shade - Existing buildings can provide shade for adjacent playgrounds - Shade sails provide shade for smaller, high-use play areas
Temperature	- Very high or low temperatures decrease thermal comfort - Must account for air, surface, and equipment temperatures for safe play - Hot surfaces increase heat load on children, and thus risk of heat illness	- Large-scale ambient air temperature is difficult to modify, but can be influenced by design via shade, vegetation, materials, and colours - Vegetation and water can help to cool air
Airflow	- Wind flow supports thermal comfort in the warm season through evaporation of sweat and convective cooling of skin; stagnant air reduces comfort - Cold season wind increases wind chill, lowering comfort	- Wind surveys help to determine likely wind directions and speeds (i.e., windrose) - Focus on supporting warm season wind ventilation, while providing cold season wind blocking
Relative humidity	- High humidity impedes cooling through sweat evaporation, decreasing thermal comfort	- As with air temperature, it is difficult to change ambient humidity - Increasing airflow can aid in the evaporative process and vapour mixing, thus supporting comfort in the warm season
Water access	Although not a principal environmental factor related to thermal comfort per se, access to water provides an opportunity for drinking and cooling during play. It also allows for the introduction of water play options, and enhances maintenance capabilities, including the watering of trees, vegetation, and gardens—all of which affect the above environmental factors	

English: https://www.scc.ca/en/about-scc/publications/general/thermally-comfortable-playgrounds

French: https://www.scc.ca/fr/notre-organisme/publications/general/le-confort-thermique-des-terrains-de-jeu

The report advocates that addressing thermal comfort within playgrounds also mitigates environmental health risks for children. Shade, both natural and from man-made structures, is a significant factor in reducing surface temperatures, thereby reducing heat stress in children and minimizing skin damage risk from solar exposure. The long-term effects of recommended design changes, such as reducing solar radiation to mitigate skin cancer, will not immediately be known. However, studies have shown demonstrable reductions in UV exposures during play if shade is readily available (Vanos et al., 2017a, b).

The report, which is freely available on SCC’s website in English https://www.scc.ca/en/about-scc/publications/general/thermally-comfortable-playgrounds and French https://www.scc.ca/fr/notre-organisme/publications/general/le-confort-thermique-des-terrains-de-jeu, is intended to reach a broad, public audience and raise awareness about hazardous thermal conditions at playgrounds.

### Informational annex on thermal comfort integrated within CAN/CSA-Z614:2020

The NPPS developed the informational annex to provide useful design suggestions for designing thermally comfortable playgrounds. The target audience for the annex are designers, planners, and safety inspectors—professionals who regularly utilize the standard. The motivation to include the annex within the standard is to enhance awareness of thermal comfort issues and design solutions. The information in the annex is intended for use during the planning stages of playground development, throughout the maintenance period, or when renovating existing playgrounds, and is applicable to the various geographic and climate zones of Canada. The technical committee agreed by consensus to include the informative annex on thermal comfort in the 2020 edition of the CAN/CSA-Z614 standard.

### Integrating thermal comfort considerations within a pan-Canadian School Ground Greening Design Competition

Tree Canada, a national non-profit organization, runs an annual program entitled *Greening Canada’s School Grounds* which awards grants to help schools green their grounds. Schools develop proposals to plant and nurture trees around their schoolyards and increase green space; many of the proposals highlight nature-based outdoor learning experiences for students. Health Canada collaborated with Tree Canada to integrate thermal comfort in the Fall 2019 edition of the competition. Tree Canada’s online application form asked applicants to address thermal comfort and climate change within their application. Information on thermally comfortable design was provided to applicants to assist them, including recommendations from the *Thermally Comfortable Playgrounds* technical report.

## Implications

Thermally safe and comfortable playgrounds are critically needed to help communities across Canada increase the time children can safely play outdoors. Recognizing the importance of environmental health hazards, climate change, and the long-term consequences of these issues, multiple organizations and levels of government supported an evidence-based project to help ensure playgrounds in Canada are designed to be thermally comfortable in all environmental conditions.

Standards and guidance documents have traditionally focused on basic, acute injury prevention (such as safety and structural integrity of equipment), as well as maintenance, accessibility, and design for supervision. The topic of thermal comfort at playgrounds is much less widely understood. For example, out of the 141 applications received in Tree Canada’s 2019 *Greening Canada’s School Grounds*, 136 applicant schools addressed the questions on climate change and thermal comfort criteria. However, a review of the applications showed it was evident that the topic was new to many schools, and applicants would benefit from additional information, educational support, and guidance. The expert surveys confirmed that playground designers give a higher prioritization to design issues included in CAN/CSA-Z614, as compared with those not addressed within the standard. Therefore, it is hoped that the inclusion of the thermal comfort annex within the standard will raise awareness of the topic and lead to real changes to playground designs across Canada.

The technical report identified the need for comprehensive research to assess the health impacts of children’s exposures to extreme temperatures, solar radiation, and air pollution within playground environments. Future research could evaluate how the Thermal Comfort Annex has been used by playground designers and assess whether the standards help improve the health and safety of children in hot summer months. Quantification of various factors—such as monitoring surface temperature reductions, activity level increases, or frequency and duration of use changes—would expand knowledge of practices that moderate environmental exposure, and help identify which are most impactful by season and climate type, and how these factors affect duration and frequency of playground use.

Communication materials and activities could help to raise awareness of thermal comfort strategies and increase practical uptake. Materials could highlight the co-benefits of thermal comfort strategies, for example, by sharing how trees not only improve thermal comfort but can also help adapt to climate change, increase biodiversity, and reduce storm water overflows. Future work could adapt standards and guidelines for use in visual communication tools such as infographics to help reach a variety of audiences, including those of different ages, cultures, professions, and languages.

## Lessons learned

This project underscores the importance of standards and guidelines to promote awareness and provide practical design advice that can help communities prepare for increasing temperatures expected in many parts of Canada due to urban growth and climate change. The following key lessons learned emerged through the process of developing the guidelines:

1. *Pilot projects help raise awareness and build expertise, while providing valuable opportunities for policy makers in higher levels of government to better understand the issues.* The City of Windsor’s practical measures to address thermal comfort within their playgrounds (e.g., planting trees and installing water fountains and splash pads) helped increase physical activity levels and protect the health of children. The project also provided contextual insight to federal government employees who worked to integrate what they learned into a national standard that could be used by communities across Canada.
2. *The involvement of subject matter experts in collaboration with existing technical committees results in stronger and more holistic recommendations.* The CSA Group Technical Committee is comprised of people with diverse professional backgrounds. The desire to expand the standard beyond traditional issues required the insight of external playground safety experts, atmospheric scientists, biometeorologists, child health experts, and others. The diversity of professionals involved in this project helped reveal important considerations and contextual knowledge from various practical disciplines to aid in the development of more complete guidance.
3. *Standards and guidance are essential to maintain uniform compliance and raise awareness.* While environmental safety considerations have seen increased attention due to the growing concerns of climate change, addressing these issues is challenging because of a lack of available guidance. Low levels of awareness of thermal comfort concepts limit uptake by playground designers relative to traditional injury prevention and safety issues. In addition to the thermal comfort annex within the standard, the technical report is freely available to raise awareness of these issues.
4. *The establishment of design guidance for addressing environmental hazards such as extreme heat is needed to help communities across Canada prepare for climate change.* Many communities across Canada are projected to experience an increasing number of hot days throughout the summer. Building awareness of summertime environmental considerations—such as sun protection, shade, water access, and ventilation—for creating thermally safe and comfortable spaces will result in playgrounds and outdoor spaces that children and visitors can use safely into the future under different climate scenarios.

## Conclusion

Children are physiologically vulnerable to environmental extremes. Given the hotter summers in many parts of Canada from climate change, it is increasingly important to plan and design thermally comfortable outdoor spaces. While this project focused on developing guidance for designing thermally safe playgrounds in the Canadian context, many of the principles universally apply to playgrounds in other parts of the world for all seasons. It is hoped that this Canadian example could help spur additional research and action to improve thermal comfort in playgrounds as well as inspire the production of similar and expanded guidelines and standards in other countries.

## Implications for policy and practice

What are the innovations in this policy or program?

- This project developed innovative ideas for integrating microclimate concepts into playground designs.
- Existing Canadian and international standards for children’s playground equipment and surfacing focus exclusively on reducing acute injuries.
- With climate change, many parts of Canada could experience more frequent hot days (>30°C), which could increase heat stress in children during play.
- A Canadian playground safety standard was updated to include guidance on how to design for thermal comfort by increasing shade, vegetation, and water features.

What are the burning research questions for this innovation?

- Many of the health benefits expected from the development of thermally comfortable playgrounds are long-term in nature; therefore, it is imperative to develop new ways to assess the effectiveness of these practices in order to promote adoption.
- It is important to develop communication materials tailored to different users to raise awareness of strategies included in the thermally safe playground guidelines and increase their practical uptake.
- Further research is needed to evaluate which playground design factors maximize children’s physical activity levels and improve the health of children under a range of weather conditions.
